# Spatial Distribution of Soil Organic Carbon and Total Nitrogen Based on GIS and Geostatistics in a Small Watershed in a Hilly Area of Northern China

**DOI:** 10.1371/journal.pone.0083592

**Published:** 2013-12-31

**Authors:** Gao Peng, Wang Bing, Geng Guangpo, Zhang Guangcan

**Affiliations:** 1 Shandong Agricultural University, College of Forestry/Taishan Mountain Forest Ecosystem Research Station/Shandong Provincial Key Laboratory of Soil Erosion and Ecological Restoration, Tai'an, Shandong, P.R. China; 2 Research Institute of Forest Ecology Environmental Protection, Chinese Academy of Forestry, Beijing, P.R. China; DOE Pacific Northwest National Laboratory, United States of America

## Abstract

The spatial variability of soil organic carbon (SOC) and total nitrogen (STN) levels is important in both global carbon-nitrogen cycle and climate change research. There has been little research on the spatial distribution of SOC and STN at the watershed scale based on geographic information systems (GIS) and geostatistics. Ninety-seven soil samples taken at depths of 0–20 cm were collected during October 2010 and 2011 from the Matiyu small watershed (4.2 km^2^) of a hilly area in Shandong Province, northern China. The impacts of different land use types, elevation, vegetation coverage and other factors on SOC and STN spatial distributions were examined using GIS and a geostatistical method, regression-kriging. The results show that the concentration variations of SOC and STN in the Matiyu small watershed were moderate variation based on the mean, median, minimum and maximum, and the coefficients of variation (CV). Residual values of SOC and STN had moderate spatial autocorrelations, and the Nugget/Sill were 0.2% and 0.1%, respectively. Distribution maps of regression-kriging revealed that both SOC and STN concentrations in the Matiyu watershed decreased from southeast to northwest. This result was similar to the watershed DEM trend and significantly correlated with land use type, elevation and aspect. SOC and STN predictions with the regression-kriging method were more accurate than those obtained using ordinary kriging. This research indicates that geostatistical characteristics of SOC and STN concentrations in the watershed were closely related to both land-use type and spatial topographic structure and that regression-kriging is suitable for investigating the spatial distributions of SOC and STN in the complex topography of the watershed.

## Introduction

As important factors in global biogeochemical cycling of the terrestrial ecosystem, soil organic carbon (SOC) and total nitrogen (STN) are important in alleviating global warming, mitigating land degradation, improving food security, and enhancing crop production [1], [2], [3]. They are also at the heart of the global carbon-nitrogen cycle and climate change research. SOC and STN are carbon and nitrogen sources for plant growth. They also affect soil biodiversity and the structure and physical stability of soil that enables it to resist erosion [4]. SOC and STN have strong spatial heterogeneity, with internal changes in the vertical and horizontal directions and external exchanges with the atmosphere and biosphere [5]. Many factors, such as topography, land-use type, field management and vegetation, can control SOC and STN spatial variability at various scales. Understanding and incorporating such heterogeneity and spatial distribution characteristics can improve the precision of carbon-nitrogen budgets and assist in implementation of effective measures toward vegetation recovery.

There is considerable research into SOC and STN spatial distributions on different scales [6], [7], [8], [9], [10], and results show that SOC and STN have a changing continuum with a non-uniform spatial distribution. Several studies have shown variability in topography, vegetation, cultivation, land use and parent material [11], [12], [13], [14].

However, current research mainly relies on the ordinary kriging method, which produces large uncertainty in the prediction of soil spatial distribution because of the impacts of land use, topographic characteristics and other factors [15]. In recent years, some methods have been proposed to solve this problem, such as the regression-kriging method, the geographically weighted regression method (GWR) and the geographically weighted regression kriging method (GWRK) [16], [17]. However, among these methods, only the regression-kriging method can incorporate topographic factors, vegetation coverage and other elements and thereby improve the accuracy of spatial prediction [18], [19], [20].

Some researchers have studied SOC and STN spatial variability in different regions of China, including the black-soil region in the northeast and the Loess Plateau in the northwest [21], [22], [23]. However, there is generally little quantitative information on this spatial variability on small-watershed scales in the rocky mountain area of the northern part of the country. This is especially true regarding the few spatial variability studies using regression-kriging and geographic information system (GIS) technology, limiting the capability of evaluating the carbon budget and predicting the ecosystem response to environmental and climate change.

In this paper, we selected the Matiyu small watershed, which is typical of the rocky mountain areas of northern China, as a research site. We used regression-kriging and GIS to achieve the following objectives: 1) to reveal and analyze spatial SOC and STN distributions at the scale of a small watershed; 2) to address the impacts of different land-use types, elevation, vegetation coverage, and other factors on those spatial distributions; and 3) to compare SOC and STN prediction accuracy using regression-kriging and ordinary kriging methods.

## Materials and Methods

### Ethics Statement

The research station for this study is managed by Shandong Agricultural University. The study was approved by the Mountain Tai Forest Ecosystem Research Station of the State Forestry Administration.

### Study area condition

**Figure 1:**
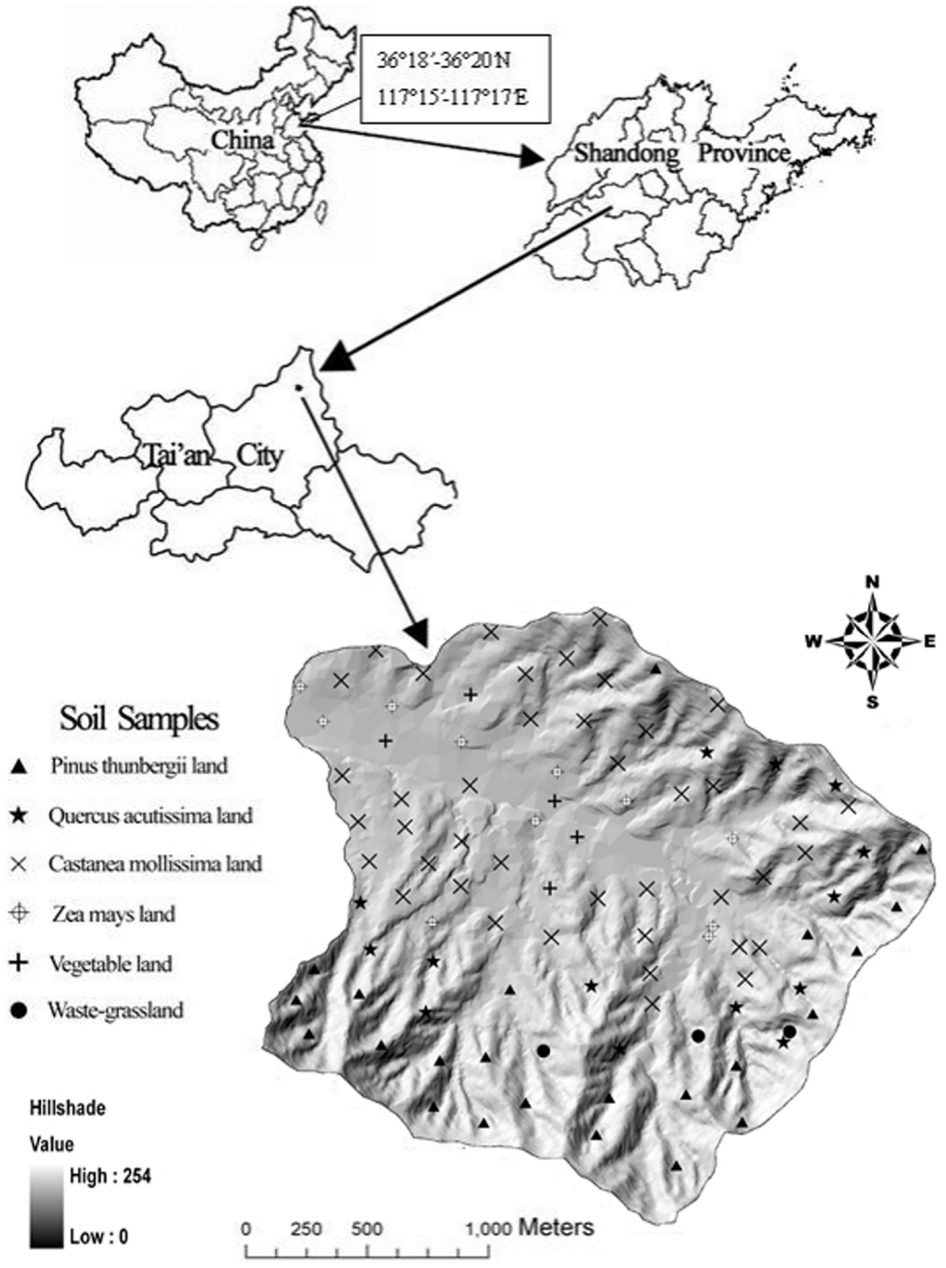
Distribution map of soil sample points (0–20 cm) in Matiyu small watershed.

The Matiyu watershed is in Tai'an city, Shandong Province. It has an area of 4.2 km^2^ and lies between 36°18′–36°20′N and 117°15′–117°17′E (Figure 1), with elevations ranging from 219 m to 595 m. The climate is semiarid, with an average of 765 mm of annual precipitation; 70% of this precipitation falls from June through September. Annual average air temperature is 12.8°C, the accumulated temperature greater than 10°C is 4120°C, and there are 200 frost-free days.

The dominant soil type is cinnamonsoil. The study area includes the following major land-use types: forest (including *Pinus thunbergii*, *Quercus acutissima*, and *Castanea mollissima*), farmland (including *Zea mays* and vegetables), and waste-grassland.

### Sampling and measurement methods

The soil sampling design was based off of a digital topographic map of the watershed at a scale of 1∶10000, which was used to generate a raster digital elevation model (DEM) map with a resolution of 10 m×10 m. A grid square crossed by the study boundary was regarded as a distinct unit if more than half of it lay inside the study area; otherwise, it was merged into a neighboring grid. A Real Time Kinetic – Global Positioning System (RTK-GPS) receiver was used to locate each grid center in the field and to record latitudes, longitudes and elevations of sampling points. The terrain information was then recorded in detail. Within a radius of approximately 20 cm at each site, four separate sub-samples were taken from the top 20-cm of soil using a 5.0-cm diameter soil auger. These samples were then mixed and were considered representative of the soil at the site.

In total, 97 soil samples (0–20 cm) were collected in Matiyu watershed during October 2010 and October 2011 (Figure 1) [24], [25]. Soil samples were air-dried for approximately 7 days and then passed through a 0.25-mm sieve. SOC and STN contents were determined in duplicate for each sample, using the potassium dichromate oxidation and micro-Kjeldahl methods [26].

### Acquisition of topographic factor attributes and NDVI

Topographic factors were computed using a spatial analysis model and digital topography analysis, and included elevation (H), slope (β), sinα and cosα of aspect, compound topographic index (CTI), stream power index (SPI), and sediment transport index (STI). The normalized difference vegetation index (NDVI) was acquired from a Landsat 5 image from August 30, 2010.

### Interpolation of regression-kriging

**Figure 2:**
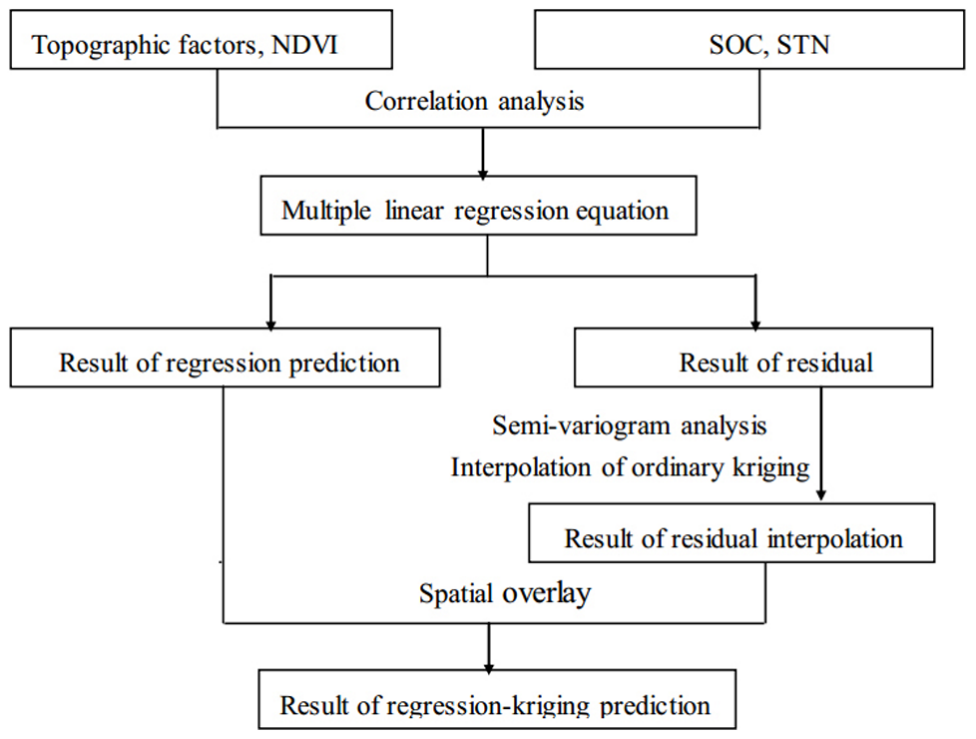
Flowchart of regression-kriging.

Figure 2 shows a flowchart of the regression-kriging method. Taking into account the spatial distribution law and geographic influence factors, the method simulates both spatial distribution trends and uncertainty. Equations were established between the assistant variable and target variables, and then ordinary kriging was used to separate the trend item and interpolate the residual. Finally, the spatial overlay was carried out between the trend item of regression prediction and the residual value of ordinary kriging to obtain the predicted value of the target variable [27], [28].

### Precision evaluation of prediction results

Twenty-six of the 97 soil samples were randomly extracted from the data to test the model's predictive accuracy based on data from the remaining 71 soil samples. Prediction accuracy was evaluated by comparing observed and predicted values of SOC and STN from validation point locations. Mean prediction error (*MPE*) and root mean square prediction error (*RMSE*) were selected as evaluation indexes, and *R*_I_ was used to analyze improvement of the prediction accuracy by comparing regression-kriging with ordinary kriging [29], [30].
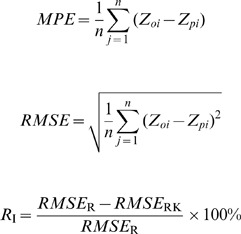
where *MPE* is the mean prediction error; *RMSE* is the root mean square prediction error; *n* represents sampling points of the test dataset; Z*_oi_* and Z*_pi_* are observed and predicted values of the sampling points, respectively; and *R*_I_ is the improvement of prediction accuracy from comparing regression-kriging with ordinary kriging. If *R*_I_ is positive, it means that prediction accuracy of regression-kriging was higher than that of ordinary kriging, and vice-versa for a negative *R*_I_ value. *RMSE*_R_ is the root mean square prediction error of ordinary kriging, and *RMSE*_RK_ is that of regression-kriging.

#### Software platform

SPSS17.0 software was used for classical statistical and regression analyses of SOC and STN and for testing their differences under different land use types via ANOVA. ArcGIS 9.2 was used to establish the small-watershed DEM and extract its topographic factors. GS+7.0 was used for semi-variogram analysis of regression prediction and residual values. ArcGIS9.2 was then used to perform the spatial plus operation between the trend item of regression prediction and the residual value of ordinary kriging [31]. Finally, the spatial prediction distribution map of SOC and STN in the Matiyu watershed was produced.

## Results

### Descriptive statistics of SOC and STN in Matiyu watershed

Mean SOC and STN concentrations in the watershed were 11.91 g·kg^−1^ and 1.18 g·kg^−1^, respectively. Respective coefficients of variation (CVs) were 24.10% and 23.73%, respectively, and both CVs were moderate (Table 1).

**Table 1:** Statistical results of mean concentrations of SOC and STN in Matiyu small watershed^†^.

Soil nutrients‡	Mean (g·kg^−1^)	Median (g·kg^−1^)	Min.(g·kg^−1^)	Max. (g·kg^−1^)	Range (g·kg^−1^)	Std. Deviation	CV (%)
SOC	11.91	11.73	6.20	21.67	15.47	2.87	24.10
STN	1.18	1.16	0.54	2.57	2.03	0.28	23.73

“†” Numbers represent the mean of 2 years (2010–2011a). The following is the same.

“‡” SOC  =  soil organic carbon; STN  =  soil total nitrogen.

### Correlations of SOC and STN with environmental factors

Table 2 shows that both SOC and STN had highly significant positive correlations with elevation (correlation coefficients of 0.495 and 0.415, respectively). This indicates that the higher the elevation, the larger the SOC and STN concentrations. SOC and STN also had highly significant positive and significant positive correlations with slope, respectively. This reveals that the greater the slope, the larger the two concentrations. The increasing trend of SOC was much more significant than that of STN. Both concentrations had little correlation with the sine of aspect. However, SOC and STN had highly significant positive and significant positive correlations with the cosine of aspect, respectively. This cosine measures the degree of northward orientation; thus, the greater the northward orientation, the larger the SOC and STN concentrations.

**Table 2:** Correlation analysis of SOC and STN with the environmental factors.

Soil nutrients^‡^	Elevation	slope	cosα	sinα	CTI	SPI	STI	NDVI
SOC	0.495^**^	0.339^**^	0.298*	−0.052	−0.094	0.030	0.117	0.199*
STN	0.415^**^	0.249*	0.400^**^	−0.020	−0.083	0.016	0.042	0.215*

and ^**^ indicate significance at P<0.05 and P<0.01, respectively.

“‡” SOC  =  soil organic carbon; STN  =  soil total nitrogen; cosα  =  cosine of aspect; sinα  =  sine of aspect; CTI  =  compound topographic index; SPI  =  stream power index; STI  =  sediment transport index; NDVI  =  Normalized difference vegetation index.

Both SOC and STN had significant positive correlations with NDVI, which indicates that the greater the vegetation cover, the higher the two concentrations. Both SOC and STN had little correlation with CTI, SPI or STI.

### Regression-kriging models and semi-variogram analysis

Using the multiple linear stepwise regression method, all topographic factors and NDVI were used to explain the variability of SOC and STN concentrations. Equation fitting results were as follows:

*r* ^2^ = 0.330 (*P*<0.05)

*r* ^2^ = 0.328 (*P*<0.01)

The elevation and cosine of aspect that joined the prediction of SOC and STN were the optimal factors from all selected environmental factors. The determination coefficients of SOC and STN (*r* ^2^) were 0.330 and 0.328, and the equation fits were good.

**Figure 3:**
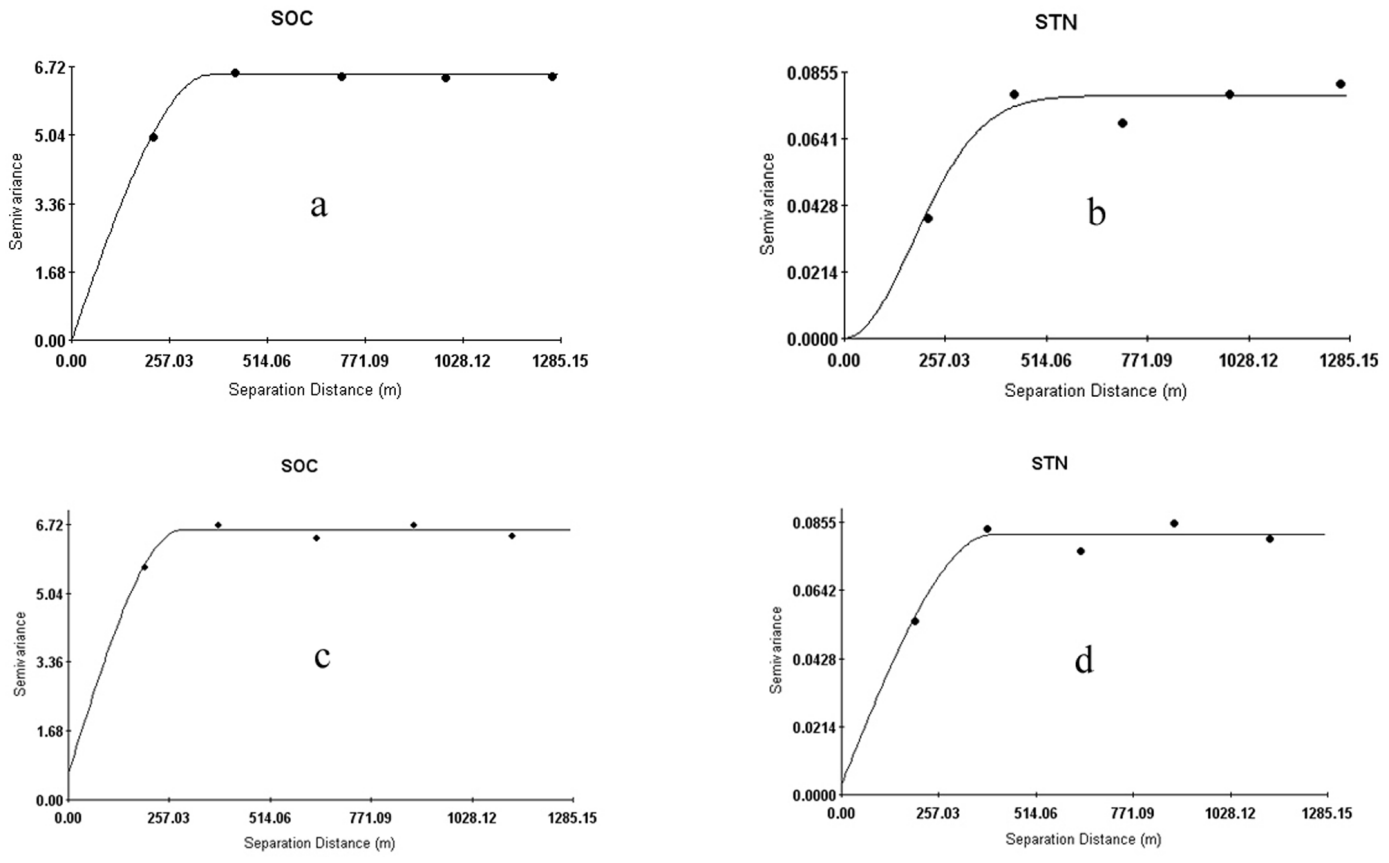
Semi-variogram analysis of SOC and STN for regression-kriging (a, b) and ordinary kriging (c, d).

Spatial autocorrelations of SOC and STN from regression-kriging (RK) and ordinary kriging (OK) were both low; however, the effect of regression-kriging was better than that of ordinary kriging, and the Nugget/Sill were 0.2% and 0.1%, respectively (Table 3, Figure 3). The semi-variogram test showed that structural factors such as topography, vegetation and climate were the primary causes of SOC and STN spatial variation; random factors had little influence.

**Table 3:** Semi-variogram analysis of SOC and STN and its parameters.

Soil nutrients‡	Data Item	Active lag distance(m)	Lag interval(m)	Model	Nugget *C*_0_	Sill *C*_0_+*C*	Nugget/Sill *C*_0_/(*C*_0_+*C*) (%)	Range (m)	*R* ^2^
SOC	residual error(RK)	1285.15	280	Spherical	0.0100	6.5230	0.2	365	0.996
STN	residual error(RK)	1285.15	280	Gaussian	0.0001	0.0779	0.1	424	0.915
SOC	raw data(OK)	1285.15	250	Spherical	0.6500	6.5740	9.9	288	0.880
STN	raw data(OK)	1285.15	250	Spherical	0.0031	0.0817	3.8	399	0.908

“‡” SOC  =  soil organic carbon; STN  =  soil total nitrogen; RK  =  regression-kriging; OK  =  ordinary kriging.

### Evaluation of prediction accuracy using different prediction methods

**Figure 4:**
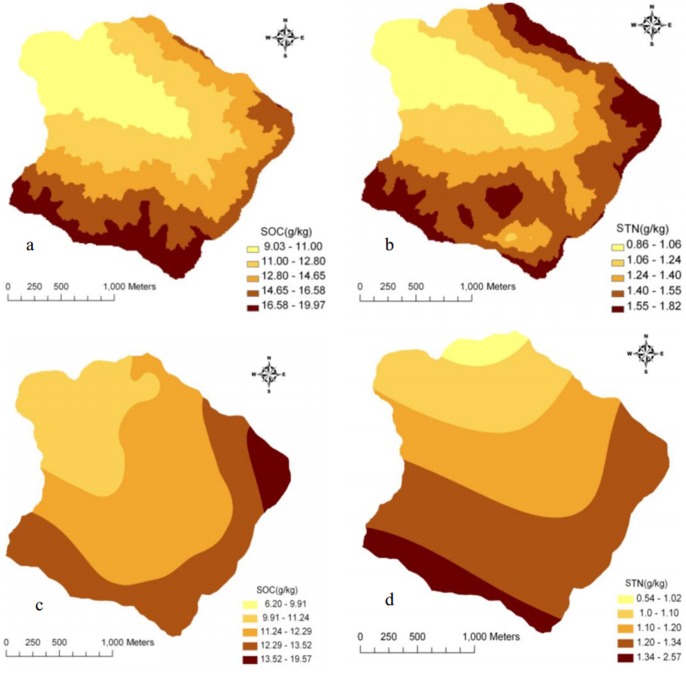
Distribution map of SOC and STN concentrations by regression-kriging (a, b) and ordinary kriging (c, d) in Matiyu small watershed.

**Figure 5:**
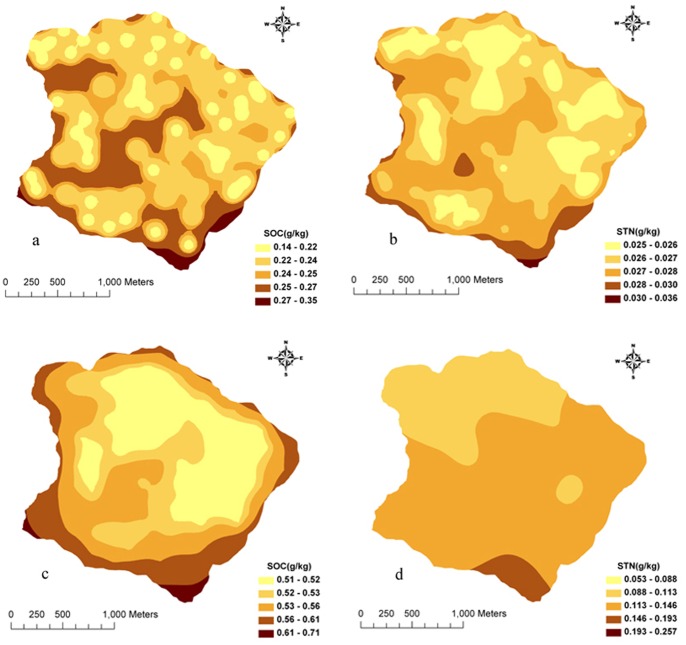
Maps of Prediction Standard Error of SOC and STN by regression-kriging (a, b) and ordinary kriging (c, d) in Matiyu small watershed.

In accordance with the flow chart of regression-kriging, linear combinations of environmental factors were considered as the external drift trend term to separate residual error and eliminate non-stationary properties to increase prediction accuracy. Seventy-one samples were randomly selected to conduct ordinary kriging interpolation for regression residual error of SOC and STN in the Matiyu small watershed. Meanwhile, as a control, those 71 samples were used to conduct ordinary kriging interpolation. From the prediction errors and distribution maps of regression-kriging and ordinary kriging (Figure 4, Figure 5), the result showed that the regression-kriging was better than that of ordinary kriging. The regression-kriging predictions were much more detailed concerning the partly variation and topographical relationships and much closer to the observed spatial distribution of SOC and STN. The other 26 samples were used to compare the accuracy of the two prediction methods (Table 4). The regression-kriging predicted values were very close to the measured ones, and its prediction accuracy was superior to that of ordinary kriging. The improvements of prediction accuracy (*R*_I_) of SOC and STN were 16.80% and 17.24%, respectively (Table 4).

**Table 4:** Comparison of prediction accuracy between regression-kriging and ordinary kriging.

Soil nutrients‡	Ordinary kriging		Regression-kriging		*R*_I_(%)
	*MPE*	*RMSE*	*MPE*	*RMSE*	
SOC	−0.5	3.63	0.39	3.02	16.8
STN	−0.06	0.29	−0.01	0.24	17.24

“‡” SOC  =  soil organic carbon; STN  =  soil total nitrogen; MPE  =  mean prediction error; RMSE  =  root mean square prediction error; RI  =  improvement of prediction accuracy.

### SOC and STN spatial distribution characteristics and their correlations in small watershed

**Figure 6:**
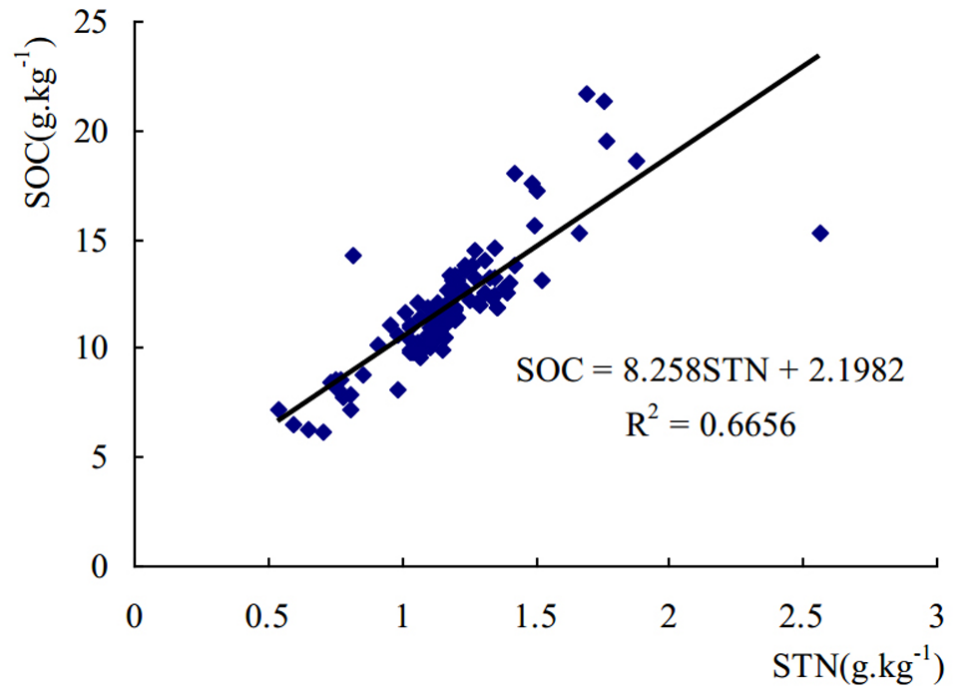
Regression equations of SOC and STN in Matiyu small watershed.

The distribution maps of regression-kriging showed that both SOC and STN concentrations decreased from southeast to northwest in the watershed, similar to the DEM results (Figure 4). SOC and STN were strongly correlated, with a correlation coefficient of 0.6656 (*P*<0.01). Regression analysis revealed that SOC and STN had a highly significant linear relationship (Figure 6).

## Discussion

### Influences on SOC and STN spatial distributions under different land use types

A change of land-use type can generate corresponding changes of microenvironment for every land type and subsequently alter soil nutrients [32], [33]. Table 5 reveals that SOC concentrations in varying land use types were ranked as *P. thunbergii* > *Q. acutissima* > vegetable > *Z. mays* > *C. mollissima* > waste-grassland. STN concentrations were ranked as *P. thunbergii* > vegetable > *Q. acutissima* > *Z. mays* > *C. mollissima* > waste-grassland. This suggests that land-use type had a significant impact on spatial SOC and STN patterns.

**Table 5:** Changes of SOC and STN concentrations from different land use types*.

Land use types	Samples	SOC(g·kg^−1^) ‡	TN(g·kg^−1^)
*Pinus thunbergii* land	14	14.07±0.57c	1.41±0.07c
*Quercus acutissima* land	23	13.05±0.93bc	1.20±0.07bc
*Castanea mollissima* land	41	10.65±0.33ab	1.03±0.03ab
*Zea mays* land	11	10.70±0.41ab	1.16±0.04abc
Vegetable land	5	13.12±1.18bc	1.37±0.08c
Waste-grassland	3	9.65±1.52a	0.93±0.15a

means within a column that contain different letters are significantly different at p<0.01;.

means within a column that contain the same letters are not significantly different at p<0.05.

“‡” SOC  =  soil organic carbon; STN  =  soil total nitrogen.

The variance analysis showed that differences of SOC and STN concentrations for varying land use types were identical (*P*<0.01), whereas differences among vegetable, *Z. mays* and *C. mollissima* lands were not significant (*P*>0.05). We speculate that these results may be attributed to the following four factors: 1) Forest land (such as *P. thunbergii* and *Q. acutissima*) could fix plentiful SOC and STN because of flourishing soil plant roots and a thicker forest litter layer, which were easily resolved and beneficial to SOC and STN accumulation; 2) Litterfall on *C. mollissima* land was often collected by humans, which reduced land cover and increased soil erosion with rainfall, and therefore, SOC and STN concentrations were lower than on other land use types (except waste-grassland); 3) Farmland, especially vegetable land, was close to residential areas and had increased application of chemical fertilizers, so SOC and STN concentrations were higher than in waste-grassland; and 4) Soil and water loss was very serious in the waste-grassland, so its SOC and STN concentrations were the lowest of all land use types.

### Influences on SOC and STN spatial distributions at different elevations and aspects

The spatial distributions of soil nutrients were correlated with many environmental factors. Some research indicates that SOC and STN concentrations had significant positive correlations with elevation, cosine of aspect, and other topographic factors [34]. However, this research was mainly on pure forest land or farmland and used the ordinary kriging method. Little work has been conducted for a small watershed with varying elevations and aspects. Table 6 again shows that SOC and STN concentrations had significant positive correlations with elevation, which is consistent with the distribution map of those concentrations (Figure 4).

**Table 6:** Changes of SOC and STN concentrations at different elevations*.

Elevation(m)	samples	SOC(g·kg^−1^)‡	STN(g·kg^−1^)
220–290	35	10.70±0.36a	1.11±0.04a
290–370	29	11.32±0.42a	1.08±0.04a
370–500	33	13.71±0.55b	1.32±0.06b

Means within a column that share different letters are significantly different at p<0.05; the same letters are not significantly different at p<0.05.

“‡” SOC  =  soil organic carbon; STN  =  soil total nitrogen.

The variance analysis showed that the effects of elevation on SOC and STN concentrations were identical. For both nutrients, the 370–500 m elevation band was significantly different from 220–290 m and 290–370 m (*P*<0.05), but 220–290 m was not significantly different from 290–370 m (*P*>0.05). We suggest that these results may be due to the following factors: 1) The sample area at high elevation (370–500 m) supported SOC and STN accumulation because it had a low average soil temperature, slow nutrient decomposition and fewer human impacts; and 2) The sample area at low elevation (220–290 m) was not beneficial for accumulation due to rapid nutrient decomposition and greater human influence.

Aspect can also modify the spatial distribution of soil nutrients [35], [36]. In the study, the aspects were divided into three classes at 90° intervals from due north; 0°–45° and 315°–360° were shady slopes, 45°–135° and 225°–315° were half-sunny slopes, and 135°–225° were sunny slopes. Table 7 shows that SOC and STN concentrations with different aspects were ranked as follows, shady slopes > half-sunny slopes > sunny slopes. These results are consistent with the distribution map of SOC and STN concentrations (Figure 1, Figure 4).

**Table 7:** Changes of SOC and STN concentrations from different aspects*.

Aspect	samples	SOC(g·kg^−1^)‡	STN(g·kg^−^1)
shady slope	36	12.06±0.50a	1.25±0.06b
half-sunny slope	44	12.03±0.45a	1.16±0.03ab
sunny slope	17	11.26±0.53a	1.08±0.05a

Means within a column that share different letters are significantly different at p<0.05; the same letters are not significantly different at p<0.05.

“‡” SOC  =  soil organic carbon; STN  =  soil total nitrogen.

The variance analysis revealed that the differences of SOC concentrations with aspect were not significant (*P*>0.05). STN concentrations on half-sunny slopes were significantly different from sunny slopes (*P*<0.05), and shady slopes were significantly different from half-sunny and sunny slopes (*P*>0.05). These findings are attributed to the following factors: 1) On sunny slopes, SOC decomposes rapidly and STN concentrations are low, the opposite of conditions on shady and half-sunny slopes; and 2) fewer samples, which caused slight changes among shady, half-sunny and sunny slopes, and little significant difference of SOC and STN. Therefore, to improve the accuracy of SOC and STN spatial distributions in the study area, more attention should be given to the influences of soil temperature and the total number of samples.

## Conclusion

1. Compared with the ordinary kriging method, improvement of SOC and STN prediction accuracies by regression-kriging was 16.80% and 17.24%, respectively. This demonstrates that regression-kriging is suitable for studying SOC and STN spatial distributions in a watershed with a complex topography.
2. The semi-variogram test of the regression-kriging models revealed intermediate spatial autocorrelations of both SOC and STN, and the Nugget/Sill were 0.2% and 0.1%, respectively. The test also showed that structural factors such as topography, vegetation and climate were the main factors causing SOC and STN spatial variation in the small Matiyu watershed.
3. The distribution maps of regression-kriging revealed that both SOC and STN concentrations decreased from southeast to northwest in the watershed, similar to the DEM trend.
4. Both land use types and elevation factors had significant influences on the spatial distributions of SOC and STN concentrations in the watershed. Aspect had a smaller but still appreciable effect.
